# CREBBP/EP300 bromodomains are critical to sustain the GATA1/MYC regulatory axis in proliferation

**DOI:** 10.1186/s13072-018-0197-x

**Published:** 2018-06-08

**Authors:** Veronica Garcia-Carpizo, Sergio Ruiz-Llorente, Jacinto Sarmentero, Osvaldo Graña-Castro, David G. Pisano, Maria J. Barrero

**Affiliations:** 1 CNIO-Lilly Epigenetics Laboratory, Spanish National Cancer Research Center (CNIO), Melchor Fernandez Almagro 3, 28029 Madrid, Spain; 2 Bioinformatics Unit, Spanish National Cancer Research Center (CNIO), Melchor Fernandez Almagro 3, 28029 Madrid, Spain

**Keywords:** Epigenetics, Bromodomains, Proliferation, Cancer

## Abstract

**Background:**

The reported antitumor activity of the BET family bromodomain inhibitors has prompted the development of inhibitors against other bromodomains. However, the human genome encodes more than 60 different bromodomains and most of them remain unexplored.

**Results:**

We report that the bromodomains of the histone acetyltransferases CREBBP/EP300 are critical to sustain the proliferation of human leukemia and lymphoma cell lines. EP300 is very abundant at super-enhancers in K562 and is coincident with sites of GATA1 and MYC occupancy. In accordance, CREBBP/EP300 bromodomain inhibitors interfere with GATA1- and MYC-driven transcription, causing the accumulation of cells in the G0/G1 phase of the cell cycle. The CREBBP/CBP30 bromodomain inhibitor CBP30 displaces CREBBP and EP300 from GATA1 and MYC binding sites at enhancers, resulting in a decrease in the levels of histone acetylation at these regulatory regions and consequently reduced gene expression of critical genes controlled by these transcription factors.

**Conclusions:**

Our data shows that inhibition of CREBBP/EP300 bromodomains can interfere with oncogene-driven transcriptional programs in cancer cells and consequently hold therapeutic potential.

**Electronic supplementary material:**

The online version of this article (10.1186/s13072-018-0197-x) contains supplementary material, which is available to authorized users.

## Background

The potential to modulate histone acetylation and its outcomes is of increasing therapeutic interest. Levels of histone acetylation are dynamically regulated by the action of histone acetyltransferases (HATs) and histone deacetylases (HDACs). Bromodomain-containing proteins are able to recognize acetylated lysines in histone tails and act as effectors of the acetylation signal [1]. Such is the case of the bromo and extraterminal domain (BET) family of bromodomain-containing proteins [2]. Members of this family like the bromodomain-containing protein 4 (BRD4) are recruited to acetylated sites of the genome and favor the recruitment of the Mediator complex and pTEFb-promoting transcriptional initiation and elongation [3].

Inhibitors that specifically block the interaction of bromodomains with acetylated residues hold therapeutic promise (reviewed in [4]). Among other therapeutic properties, pan BET bromodomain inhibitors have been described to mediate important antiproliferative effects in cancer cell lines [5, 6]. BET inhibitors cause the downregulation of oncogenes that are associated with a particular class of enhancers known as super-enhancers and characterized by very high levels of histone acetylation and, in this way, block oncogene-driven proliferation of cancer cells [7].

CREBBP and EP300 are HATs that share several conserved domains, among them are the HAT domain and a bromodomain, and likely have interchangeable roles. CREBBP/EP300 functions primarily as cofactors for a number of transcription factors. Recent evidence suggests that CREBBP and EP300 are involved in the maintenance of super-enhancers and BRD4 recruitment. First, CREBBP and EP300 are highly enriched at super-enhancers compared to regular enhancers [8] and second, EP300 is recruited by hematopoietic transcription factors to mediate histone acetylation at critical regulatory regions and support BRD4 occupancy in mouse leukemia cells 2 [9].

While the CREBBP/EP300 HAT activity has been widely investigated, less is known about the relevance of the bromodomain for their function. Recently, dual inhibitors of the bromodomains of CREBBP and EP300 have been developed [10–16]. Several biological responses to these inhibitors have been reported suggesting that they have therapeutic potential. For example, they have been described to inhibit human Th17 responses [17], modulate key inflammatory genes in primary macrophages [11] and interfere with the regulatory T cells lineage [15]. In addition, several recently developed EP300/CREBBP bromodomain inhibitors have been reported to mediate antiproliferative responses in hematologic cancer cell lines, such as acute myeloid leukemia (AML) [12, 14] and multiple myeloma [18] cell lines and AR-positive prostate cancer cell lines [16]. Importantly, EP300/CREBBP bromodomain inhibitors have been reported to interfere with relevant oncogene transcription programs such as MYC, IRF4 and AR [14, 16, 18].

CREBBP/EP300 bromodomain inhibitors hold promise for future therapeutic applications; however, the mechanism of action of these compounds is not fully understood. Here, we explore the antiproliferative properties of CREBBP/EP300 bromodomain inhibition in leukemia and lymphoma cell lines and explore the molecular mechanisms responsible for such effects, using both chemical and genetic approaches. Our results show that the GATA1/MYC axis is as a key component of EP300/CREBBP bromodomain inhibitors mechanism of action in chronic myeloid leukemia (CML) cell line K562.

## Results

### CREBBP/EP300 bromodomains are critical for the proliferation of K562 cells

**Fig. 1:**
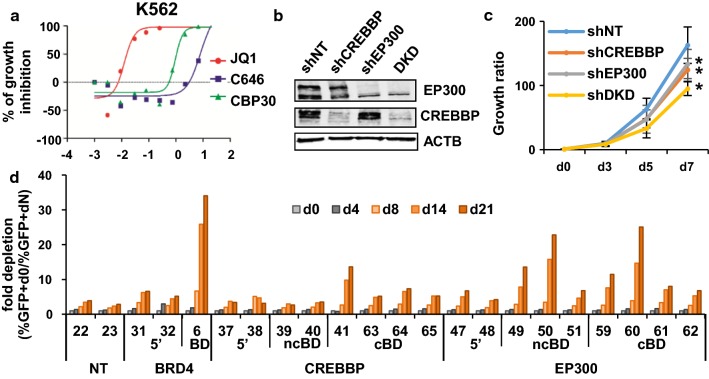
K562 are sensitive to CREBBP/EP300 inhibition. **a** Concentration-dependent (Log2 μM) growth inhibition curves of K562 cells treated with JQ1, C646 and CBP30 for 7 days. **b** EP300 and CREBBP protein levels after knock down of CREBBP or EP300 or both in K562. **c** Growth curves of K562 knock down for CREBBP or EP300 or both. *p value < 0.05 **d** Growth competition assays with cells transduced with gRNAs targeting diverse domains of CREBBP, EP300 and BRD4 including 5′ coding region (5′), bromodomain (BD), non-conserved regions of bromodomain (ncBD) and conserved regions of bromodomain (cBD)

To evaluate the potential involvement of CREBBP/EP300 in proliferation, we evaluated the sensitivity of the human CML cell line K562 to the CREBBP/EP300 bromodomain inhibitor CBP30 [10], the EP300/CREBBP HAT activity inhibitor C646 [19] and as a positive control, the BET pan inhibitor JQ1 [6]. IC50 s for growth inhibition (Fig. 1a) show that K562 cells are sensitive to JQ1 (IC50 = 0.012 µM) and CBP30 (IC50 = 0.923 µM) and to a much lower extent to C646 (IC50 = 8 µM). Differences in potency found between C646 and CBP30 are in agreement with the reduced cellular potency recently described for C646 due to consumption by abundant protein and metabolite thiol sinks [20].

To confirm that K562 cells are sensitive to CREBBP/EP300 depletion, we altered the expression of these proteins using shRNA and gene editing methods. Infection of K562 cells with lentiviruses expressing shRNAs against EP300 or CREBBP or both reduced the expression of the targeted proteins (Fig. 1b). Depletion of EP300 or CREBBP alone had effects in K562 proliferation, and effects were additive when both proteins were depleted (Fig. 1c). These results suggest that EP300 and CREBBP contribute to the proliferation of K562 cells.

Bromodomains are attractive targets due to their druggability [21]. However, it is also known that a potential complication of bromodomain inhibitor development is promiscuity among different bromodomains. Despite the fact that CBP30 has been described to have good selectivity over other bromodomains [17], we considered pertinent to confirm the involvement of EP300 and CREBBP bromodomains in the proliferation of K562 cells using an alternative method. For that, we used a recently reported method to infer the functional importance of individual protein domains of interest using the CRISPR-Cas9 genome editing technology [22, 23]. This method is based on the fact that one-third of randomly introduced mutations will result in in-frame mutations and generate a full-length protein with mutations in the particular domain targeted by the gRNA. If a domain is relevant for proliferation, more pronounced antiproliferative effects will be observed when targeting that domain than an irrelevant domain. Therefore, we interrogated the effect of introducing mutation in the EP300 and CREBBP bromodomains compared to other domains in growth competition assays (Fig. 1d and Additional file 1: Fig. S1). As expected introducing mutations in EP300 and CREBBP bromodomains caused antiproliferative effects when compared to mutations introduced in the 5′ coding region. In agreement with a role of the EP300/CREBBP bromodomains in proliferation effects were more conspicuous when targeting conserved regions of the bromodomains.

### CREBBP/EP300 bromodomain inhibitors affect the expression of super-enhancer-associated genes and genes with high levels of EP300 occupancy

**Fig. 2:**
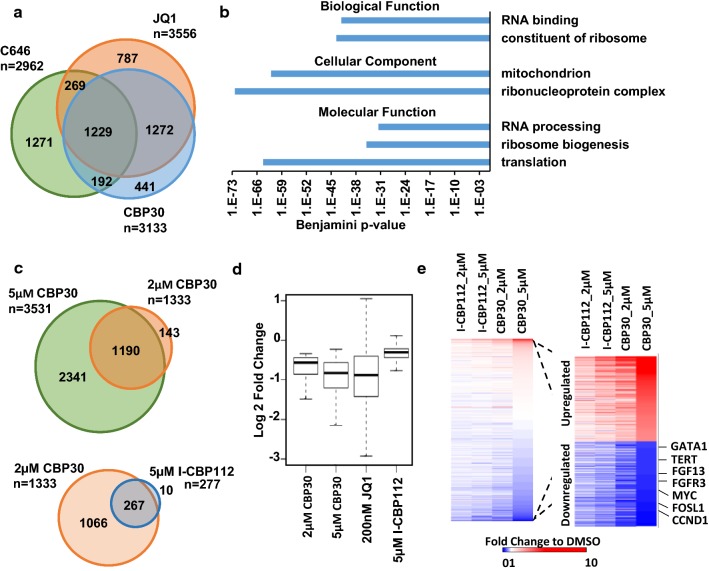
Transcriptional profiles of K562 cells treated with JQ1, CBP30 and C646. **a** Overlap of downregulated genes caused by the treatment of K562 with 200 nM JQ1, 5 µM CBP30 and 10 µM C646 for 48 h. **b** Gene ontology of commonly downregulated genes showing enrichment in the indicated categories. **c** Overlap of genes downregulated in K562 by CBP30 at 2 and 5 μM after 48 h of treatment. **d** Log2 of the fold change to DMSO caused by the indicated treatments on genes commonly downregulated by 5 µM and 2 µM CBP30. **e** Heat map on the left represents the fold change of all genes comparing the indicated treatments to DMSO. On the right, the fold change of top 400 genes changing expression is shown. Genes have been ranked according to fold change in cells treated with 5 µM CBP30

To evaluate the changes in gene expression that might be contributing to the antiproliferetive phenotype caused by CREBBP/EP300 inhibition, we carried out RNAseq analysis of K562 cells treated with 200 nM JQ1, 5 µM CBP30 and 10 µM C646. Since all treatments are related to acetylation, which is involved in gene activation, we considered that downregulated genes are more likely to be direct targets of the action of the inhibitors. About one-third of downregulated genes are shared by all treatments (Fig. 2a and Additional file 2: Table S2), suggesting that there is a functional overlap between them. Gene ontology analysis shows that shared downregulated genes are involved in RNA processing and translation that might have consequences for cell viability (Fig. 2b).

CBP30 has been reported to have 34-fold selectivity for CREBBP/EP300 over BRD4 [17]. To rule out that the CBP30 treatment at 5 µM might be mediating its effects through BRD4 inhibition, we treated K562 cells with 2 µM CBP30, a concentration reported to be unlikely to affect the BET family in vivo [17]. About one-third of genes significantly downregulated at 5 µM were also downregulated at 2 µM CBP30 treatment (Fig. 2c). As expected, these genes were more dramatically downregulated by the 5 µM treatment than the 2 µM CBP30 (Fig. 2d). There was also a significant overlap (p < 2.73 × 10^−267^) of genes downregulated by 2 μM CBP30 and the more recently described but less potent CREBBP/EP300 bromodomain inhibitor I-CBP112 [12] (Fig. 2c, d). Comparison of all genes transcriptional changes suggests a good overlap of responses to CBP30 and I-CBP112 (Fig. 2e). Genes downregulated by both 5 μM and 2 μM CBP30 were also downregulated by JQ1 (Fig. 2d) suggesting a certain degree of overlap between treatments. However, the fact that the effects of JQ1 on these genes are more heterogeneous compared to the CBP30 treatments (Fig. 2d) suggests that CBP30 and JQ1 mediate their effects through different targets.

**Fig. 3:**
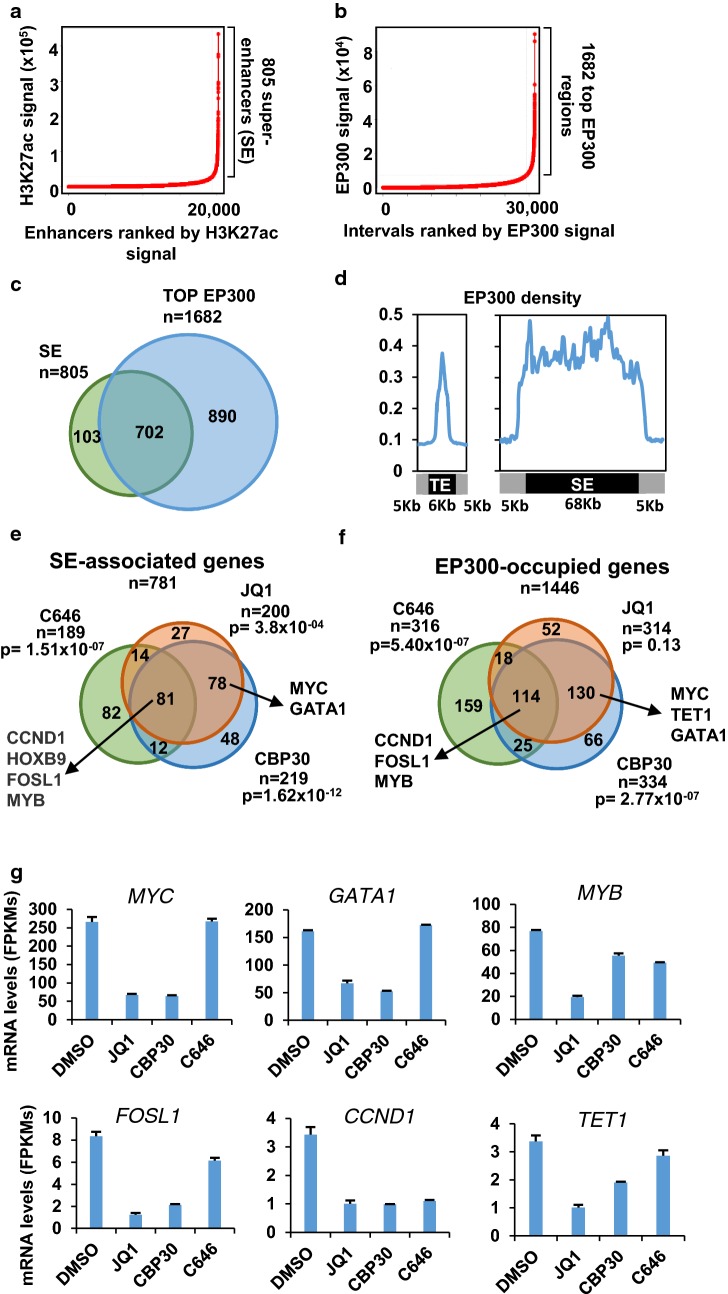
Genes associated with SE and/or occupied by EP300 are downregulated by CBP30 in K562. **a** Ranking of H3K27ac signal at genomic intervals marked with H3K27ac identifies super-enhancers in K562. **b** Ranking of EP300 signal at genomic intervals occupied by EP300 identifies top EP300 regions. **c** Overlap of genes associated with SE and top EP300 levels **d** Metagene representation of the mean of EP300 signal in regular enhancers (RE) and super-enhancers (SE). Mean size of regular enhancers and super-enhancers is shown. A 5 kb flanking region surrounding each enhancer region is also shown. **e** Overlap of genes downregulated by the 200 nM JQ1, 5 µM CBP30 or 10 µM C646 for 48 h and associated with super-enhancers. *P* value for the enrichment of super-enhancer associated genes in downregulated genes for each treatment is shown. **f** Overlap of genes downregulated by the individual treatments and occupied by top levels of EP300. *P* values for the enrichment of EP300 occupied genes in downregulated genes for each treatment is shown. **g** mRNA levels according to RNA-seq of selected genes affected by the CBP30 treatment and that are associated with super-enhancers and are bound by top EP300 levels, after treatment of K562 with 200 nM JQ1, 5 µM CBP30 and 10 µM C646 for 48 h

Next, we investigated the correlation of the genome-wide distribution of H3K27ac and EP300 with the changes in gene expression caused by the different treatments. Since JQ1 has been described to downregulate the expression of genes associated with super-enhancers [7], we used the software ROSE [7, 24] to identify super-enhancers in K562 according to the H3K27ac signal (Fig. 3a). This analysis identified 805 super-enhancers that were mapped to 781 genes. We also used ROSE to identify genomic locations with top levels of EP300 (Fig. 3b). A total of top 1682 intervals were identified which were mapped to 1446 genes. Most super-enhancers contained top levels of EP300 (Fig. 3c), suggesting that EP300 might contribute to the high levels of acetylation found at super-enhancers in K562. EP300 signal density was high at regular enhancers and more conspicuously dense at super-enhancers (Fig. 3d). These results are in agreement with a previous report that describes that EP300 is enriched in super-enhancer regions in mouse embryonic stem cells [8].

We next analyzed the overlap of genes downregulated by each treatment and associated with super-enhancers (Fig. 3e) or with top EP300 occupancy (Fig. 3f). *P* values for the enrichment of downregulated genes by each treatment and presence of super-enhancers and occupancy by top levels of EP300 shows that all three treatments caused downregulation of genes associated with super-enhancers but only CBP30 and C646 inhibitors caused the downregulation of genes occupied by top EP300 levels. This data suggest that all treatments were mediating their effects through histone acetylation but only CBP30 and C646 were mediating their effects through EP300. In addition, we confirmed enrichment of SE-associated genes and genes with top levels of EP300 in genes downregulated by I-CBP112 and CBP30, but not in genes upregulated by these treatments (Additional file 2: Fig. S2A). We notice that genes encoding important transcription factors like *MYC*, *GATA1* and *MYB* that are highly expressed in K562, associated with super-enhancers, prominently bound by EP300 and downregulated by JQ1 and CBP30 treatments (Fig. 3g). Other genes of interest like *TET1*, *FOSL1* and the cell cycle regulator *CCND1* are expressed at lower levels and downregulated by the CBP30, C646 and JQ1 treatments and are potential target genes of the above-mentioned transcription factors.

### CREBBP/EP300 bromodomain inhibitors downregulate the expression of GATA-1 and GATA-1-target genes

**Fig. 4:**
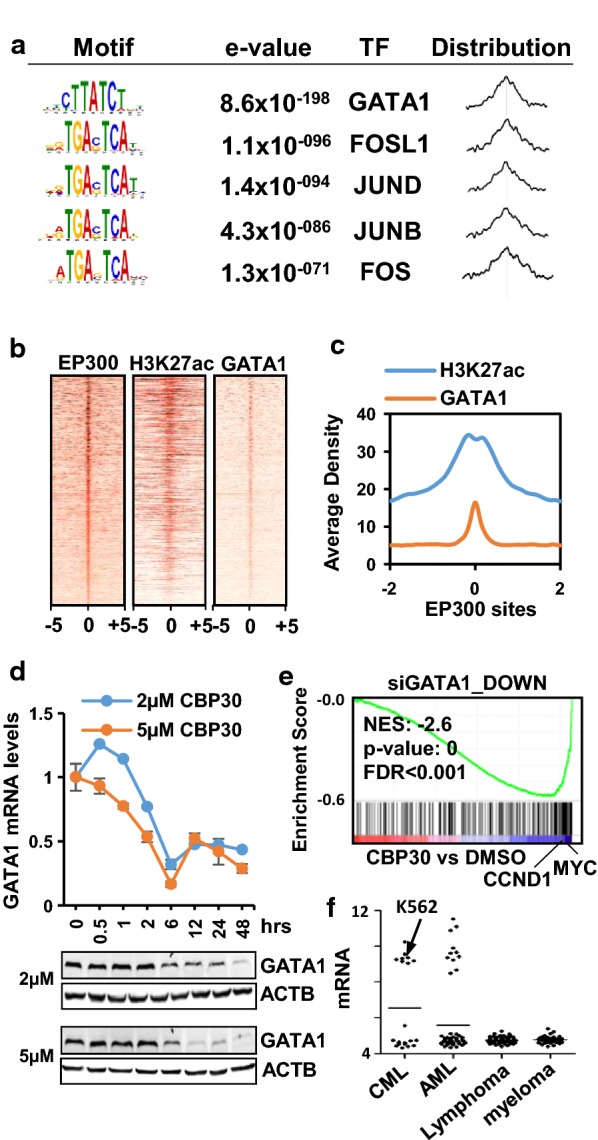
EP300 binding sites are co-occupied with GATA1 in K562. **a** Motif search in 400-bp regions centered on the constituents of top EP300 regions (n = 16,369) and predicted transcription factor binding using MEME Chip. The distribution of each motif relative to the EP300 peak is also shown. **b** Heat map of the density of EP300, H3K27ac and GATA1 signals at 10 Kb centered EP300 top occupied constituents and ranked according to EP300 signal. **c** Density of GATA1 and H3K27ac around 2Kbs of top EP300 binding sites constituents. **d** Levels of *GATA1* mRNA (upper panel) or protein (lower panel) at different time points after treatment of K562 with 2 or 5 µM CBP30. **e** GSEA analysis of K562 cells treated with 5 µM CBP30 for 48 h shows enrichment in a set of genes downregulated after GATA1 siRNA in K562 cells. **f** *GATA1* mRNA levels in the indicated subtypes of CCLE lines

We next interrogated which potential transcription factor might be recruiting EP300 to chromatin in K562 cells. We used the motif analysis tool MEME ChIP [25] to identify motifs at 400 bp centered peaks of the top EP300 constituent intervals. A motif that matches GATA1 binding sites was identified with a good e-value and a distribution that peaks at the center of the EP300 sites (Fig. 4a). Importantly, GATA1 has been previously described to interact and be acetylated by EP300 increasing its transcriptional activity [26]. Moreover, CREBBP can serve as a co-activator for GATA-1 [27]. To confirm co-occupancy of EP300 and GATA1 in K562, we analyzed the GATA1 ChIP-seq signal at the constituent intervals of top EP300 regions. Figure 4b shows that levels of EP300 and GATA1 correlate at the analyzed genomic locations. GATA1 shows maximum density at the center of EP300 sites and intervals with higher levels of EP300 located at the top of the density plot also have higher levels of GATA1. As expected, H3K27ac was also prominent at the center of EP300 binding sites (Fig. 4b, c). GATA1 mRNA levels reached a maximum downregulation at 6 h after treatment with both 2 and 5 µM CBP30 (Fig. 4d upper panel) and accordingly protein levels started to decrease at 6 h but showed minimum levels at 48 h (Fig. 4d lower panel). We further confirmed that CBP30 interferes with GATA1 transcriptional activity by performing Gene Set Enrichment Analysis (GSEA) [28]. Figure 4e and Additional file 1: Fig. S2B show that CBP30 and I-CBP112 cause the downregulation of genes downregulated after siRNA of GATA1 in K562 [29], including *MYC* and *CCND1*, which is consistent with the concept that the effects of CBP30 are at least in part due to defects on GATA1-mediated transcriptional activation. Importantly, according CCLE data, GATA1 is highly expressed in K562 cells as well in other CML and AML cell lines (Fig. 4f and Additional file 1: Fig. S3A). High expression of GATA1 is also detected in a subset of AML patients compared to other cancer types, according to TCGA (Additional file 1: Fig. S3B).

Interestingly, a shorter form of GATA1 (GATA1 s) that lacks the transactivation domain is expressed in K562 resulting from translation initiation at methionine 84 caused by the alternative splicing to exon 2 [30, 31] (Additional file 1: Fig. S4A). GATA1 s is frequently expressed in acute megakaryoblastic leukemia in patients with Down syndrome due to mutations in exon 2 that affect splicing and might be relevant for the development of this disease [31]. We analyzed the expression of GATA1 splicing variants in K562 and the effects of compounds in their expression (Additional file 1: Fig. S4B). Most abundantly expressed variants contained exon 2 and were downregulated by CBP30, while the variant with exon 2 skipping that gives rise to GATA1 s was expressed at low levels and was not significantly altered by CBP30. Eventually, the effects of CREBBP/EP300 bromodomain inhibitors on GATA1 s expression could be more precisely evaluated in a cell line that expresses high levels of GATA1 s.

### CREBBP/EP300 bromodomain inhibitors downregulate the expression of MYC and MYC-target genes

**Fig. 5:**
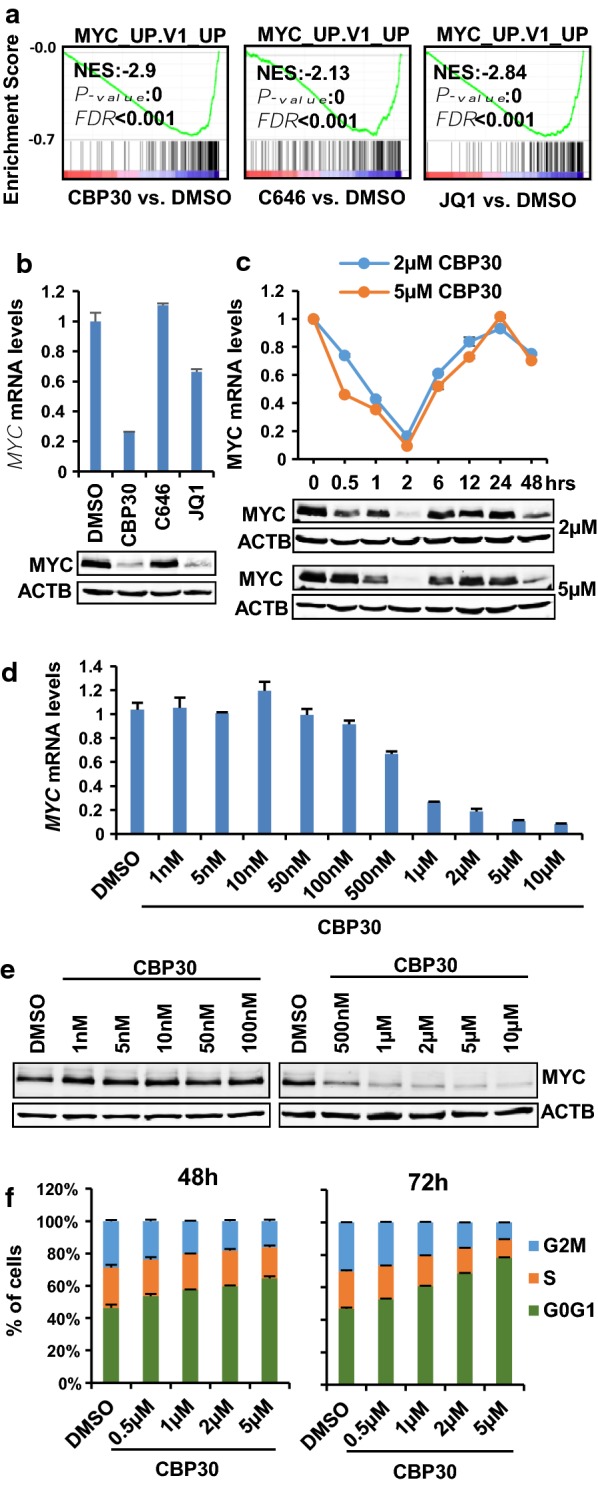
MYC and MYC transcriptional program are downregulated by CBP30 in K562. MYC and MYC transcriptional program are downregulated by CBP30 in K562.a GSEA of changes in gene expression caused by CBP30, JQ1 and C646 treatments shows enrichment in genes that are upregulated after MYC overexpression (MSigDB gene set MYC_UP.V1_UP). **b** mRNA (upper panel) and protein levels (lower panel) of K562 cells treated with 2 µM CBP30, 10 µM C646 and 150 nM JQ1 for 48 h. **c** *MYC* mRNA levels at the indicated time points in K562 cells treated with 2 µM and 5 µM CBP30. **d** *MYC* mRNA levels at 2 h after treatment of K562 cells with the indicated concentrations of CBP30 **e** MYC protein levels at 2 h after treatment of K562 cells with the indicated concentrations of CBP30. **f** Cell cycle distribution of K562 cells treated with different CBP30 concentrations for 48 or 72 h

Previous reports have demonstrated that CREBBP/EP300 bromodomain inhibitors can affect the expression of MYC [14, 18]. GSEA analysis shows that all treatments result in the downregulation of genes typically upregulated when MYC is overexpressed (Fig. 5a and Additional file 1: Fig. S2B). Treatment of K562 cells with 2 µM CBP30 for 48 h caused downregulation of MYC expression at the mRNA and protein level (Fig. 5b). C646 did not affect the levels of MYC expression (Fig. 5b) but caused the downregulation of the expression of MYC target genes (Fig. 5a), suggesting that C646 mediates its action mainly by blocking the ability of transcription factors such as MYC to stimulate transcription. We next explored the timeline of response to CBP30 regarding MYC downregulation. MYC mRNA levels were rapidly downregulated after 30-min treatment reaching a maximum downregulation at 2 h followed by a return to basal levels and a secondary decrease seen again at 48 h (Fig. 5c). Protein levels were clearly downregulated at two hours of treatment and followed similar cyclical regulation as the mRNA levels. Cyclical responses of MYC regulation have been also described in response to 4-hydroxynonenal treatment in K562 [32] although the nature of such fluctuations remains poorly understood. In order to titrate CBP30 dose response, the expression of MYC was monitored after 2-hour treatment using increasing concentrations of CBP30. At 500 nM, the levels of MYC started to decrease both at the mRNA (Fig. 5d) and protein level (Fig. 5e). Importantly, the effects in MYC expression correlated with an increase in the percentage of cells in G0/G1 after treatment with increasing concentrations of CBP30 for 48 or 72 h (Fig. 5f).

Our results suggest that the antiproliferative effects of CBP30 in K562 could be at least partially mediated by MYC, potentially in collaboration with other transcription factors. Therefore, we speculated that cell lines that express high levels of MYC could be also sensitive to CBP30. We selected lymphoma cell lines that express high levels of MYC due to amplifications (MM1S) or rearrangements (KMS11). Both MMS1 and KMS11 were sensitive to C646, CBP30 and JQ1 inhibitors (Additional file 1: Fig. S5A). In correlation with sensitivity, both CBP30 and JQ1 had significant effects on MYC expression both at the mRNA and protein levels, while C646 had more modest effects (Additional file 1: Fig. S5B). These findings are in agreement with previous reports describing the ability of EP300/CREBBP bromodomain inhibitors to modulate MYC expression in AML cell lines [14].

### CREBBP/EP300 bromodomain inhibitors displace CREBBP and EP300 from MYC-GATA1-occupied enhancers and reduce the levels of histone acetylation at these sites

**Fig. 6:**
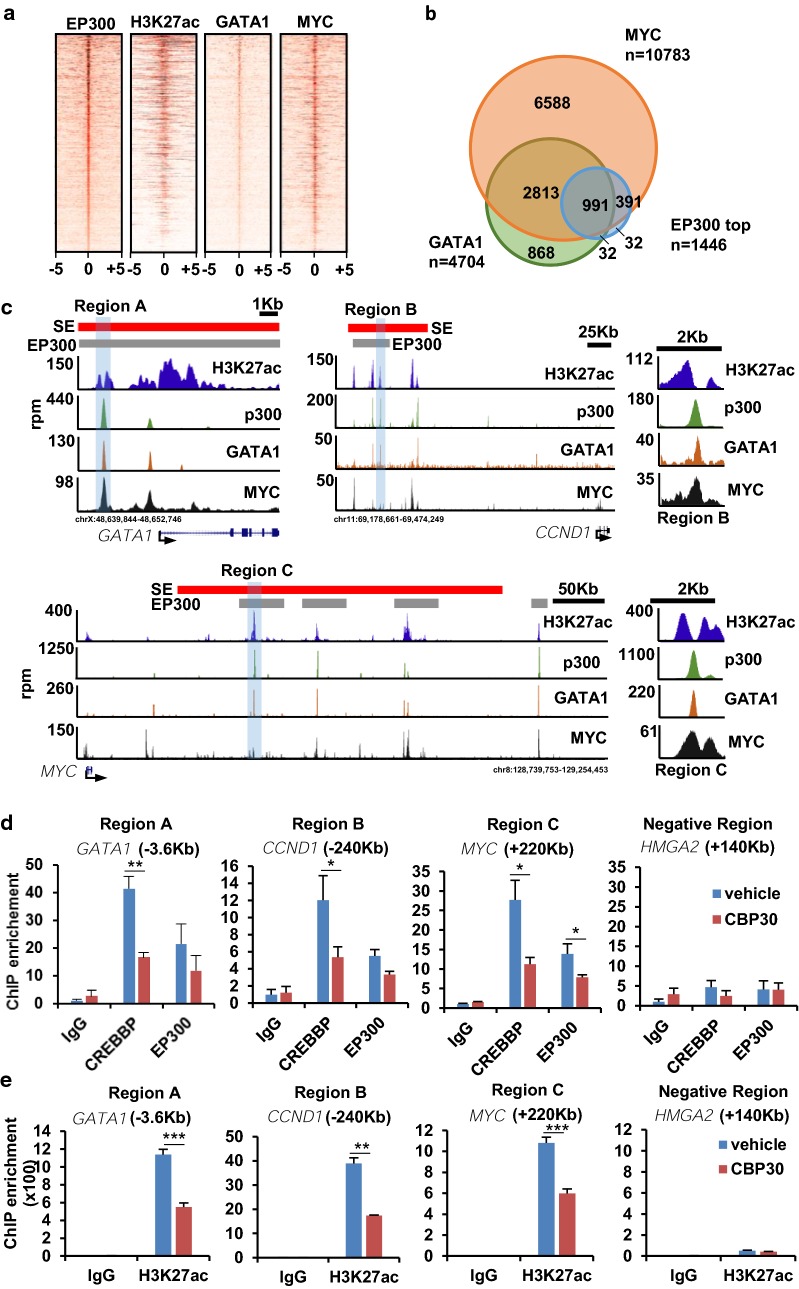
CBP30 displaces CREBBP and EP300 from chromatin at GATA1/MYC binding sites in K562 cells. **a** Heat map of the density of EP300, H3K27ac, GATA1 and MYC signals at 10 Kb centered of all EP300-occupied intervals (n = 76,018) and ranked according to EP300 signal. **b** Overlap of genes occupied by GATA1, MYC and top levels of EP300. **c** ChIP-seq profiles of H3K27ac, EP300, GATA1 and MYC at given genomic regions. Red lines show the location of super-enhancers. Gray lines show the genomic intervals with top EP300 occupancy. Regions A, B and C were used for ChIP-qPCR. Magnifications of regions B and C are also shown. **d** ChIP-qPCR showing enrichment of CREBBP and EP300 in K562 cells treated with vehicle (DMSO) and 5 µM CBP30 for one hour in regions A, B and C. Levels were normalized to the input and plotted relative to the IgGs levels in the vehicle-treated condition. The position of the amplicons relative to the transcription start site of each gene is indicated. A region downstream of the gene *HMGA2* was used as a negative control. **e** ChIP-qPCR showing enrichment of H3K27ac in K562 cells treated with vehicle (DMSO) and 5 µM CBP30 for one hour in regions A, B and C. Levels were normalized to the input and plotted relative to the IgGs levels in the vehicle-treated condition. The position of the amplicons relative to the transcription start site of each gene is indicated. A region downstream of the gene *HMGA2* was used as a negative control. *p value < 0.05, **p value < 0.005 and ***p value < 0.0005 determined by t test

Interestingly, both GATA1 and MYC-mediated transcriptional responses were affected by the CBP30 treatment in K562 cells. Density plot of GATA1 and MYC signal on EP300 binding sites suggests co-presence of both transcription factors at these sites in K562 (Fig. 6a). Most genes with top EP300 binding sites were also occupied by MYC (*p* value = 8.42 × 10^−162^) and GATA1 (*p* value = 0) (Fig. 6b). Figure 6c shows the regulatory regions of *GATA1*, *CCND1* and *MYC* containing super-enhancers (in red) and top EP300 levels (in gray). Overlapping peaks of GATA1 and EP300 appear obvious and those overlap to a lesser extend with MYC peaks and peaks of acetylation. EP300 and GATA1 peaks coincide with valleys of acetylation (see regions B and C magnifications) that likely correspond to nucleosome clearance. We next asked if CBP30 could be displacing EP300 and CREBBP from its binding sites. Importantly, we interrogated enhancer regions A and C that have been previously described to be relevant to sustain the expression of *GATA1* or *MYC* in K562 and that their disruption affects the proliferation of K562 [33]. To rule out that the CREBBP/EP300 displacement from these regions could be due to a decrease in the expression of GATA1 or MYC and consequent decrease in the occupancy of their binding sites, we conducted ChIP experiments after one hour of CBP30 treatment. At this time, protein levels of MYC and GATA1 are yet not significantly affected by the treatment (Figs. 4d, 5c). Our results show that CBP30 was able to displace EP300 and CREBBP from the interrogated genomic locations (Fig. 6d) resulting in lower levels of histone acetylation (Fig. 6e). These results suggest that the binding of the bromodomain to acetylated histones is needed to stabilize EP300 or CREBBP on GATA1/MYC binding sites and sustain histone acetylation.

### The CREBBP/EP300 catalytic inhibitor A-485 has similar effects to CREBBP/EP300 bromodomain inhibitors in K562

**Fig. 7:**
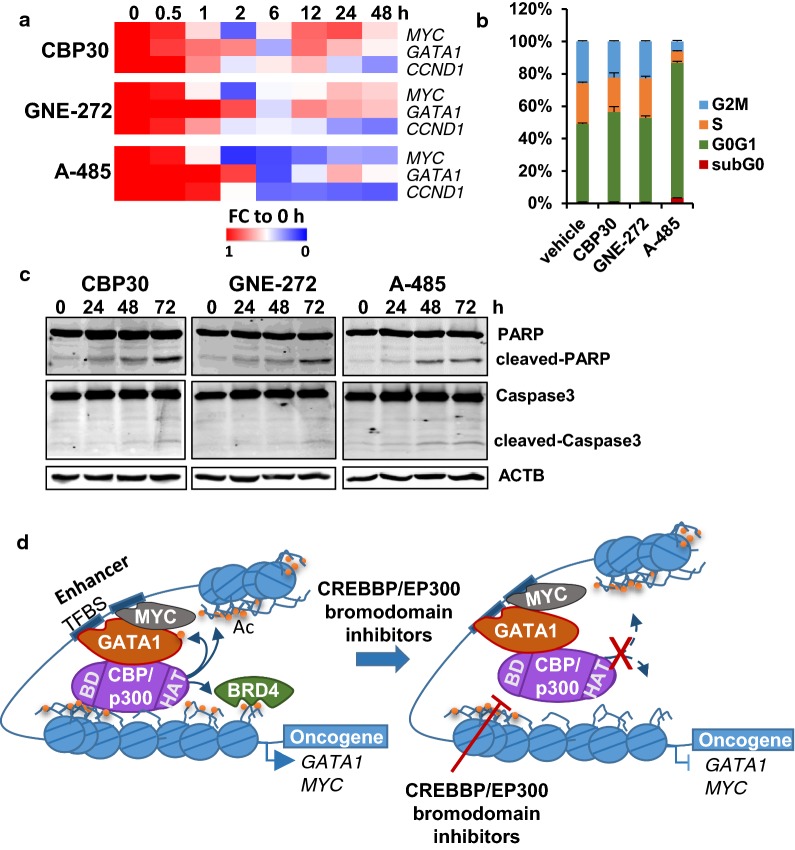
Effects of three different CREBBP/EP300 inhibitors in K562. **a** Heat map showing the changes in gene expression of *MYC*, *GATA1* and *CCND1* at different time points during the treatment of K562 cells with 2 μM of three different CREBBP/EP300 inhibitors. Fold change to d0 is represented. **b** Changes in cell cycle distribution of K562 cells treated with 2 μM of the indicated CREBBP/EP300 inhibitors for 72 h. Significant differences in G0/G1 at *p* value ≤ 0.05 were found for all compounds compared to vehicle. **c** Western blot showing the levels of PARP and Caspase3 in K562 cells treated with 2 μM of the indicated CREBBP/EP300 inhibitors and for the indicated time points. **d** Proposed model for CBP30 action. CREBBP/EP300 is recruited to chromatin by transcription factors like GATA1 or MYC that recognize binding sites in the DNA (TFBS). Properly recruited CREBBP/EP300 is able to acetylate histones. CBP30 is able to displace CREBBP/EP300 from chromatin, and therefore, acetylation of histones is reduced, which might be resulting in reduced recruitment of bromodomain-containing proteins that transduce the acetylation signal. Our model predicts that both interaction with the transcription factor and binding of the bromodomain to acetylated histones is required for proper stabilization of CREBBP/EP300 in the chromatin and acetylation of histone tails

During the course of our investigation, additional CREBBP/EP300 inhibitors have been developed by others, most remarkable the catalytic inhibitor A-485 with improved cellular potency compared to C646 [34]. We tested the sensitivity of K562 to this new inhibitor and to two recently developed inhibitors of the CREBBP/EP300 bromodomain CPI644 [15] and GNE-272 [14]. K562 cells were found to be proliferation sensitive to A-485 (IC50 = 1.18 µM), CPI644 (IC50 = 2.37 µM) and GNE-272 (IC50 = 2.6 µM). Importantly, A-485 and GNE-272 have been shown to have antiproliferative properties in xenograft mouse models without causing significant toxicity [14, 34]. Therefore, we compared the transcriptional and phenotypic effects of these two inhibitors with CBP30. Figure 7a shows that transcriptional changes in CREBBP/EP300 target genes are very similar between compounds but more dramatic changes are observed for A-485 that also correlate with more drastic effects in the cell cycle profile (Fig. 7b). The main observed effect of all inhibitors is an increase of the percentage of cells in G0/G1. In addition, after A-485 treatment a small percentage of cells (around 3%) in subG0 could be detected. This prompted us to investigate molecular events indicative of apoptosis. Figure 7c shows that after 48 h of treatment cleaved forms of PARP and Caspase3 can be detected, suggesting that some cells are undergoing apoptosis.

These results show that CREBBP/EP300 bromodomain and catalytic inhibitors have similar transcriptional and phenotypic effects in K562 and that the effects that we are observing are very likely to be on target.

## Discussion

We describe the molecular mechanisms by which CREBBP/EP300 bromodomain inhibition mediates antiproliferative effects in human CML. Our data show that inhibition of CREBBP/EP300 bromodomains can interfere with transcriptional outputs driven by oncogenes, such as GATA1 and MYC that function as transcription factors in cancer cells.

Bromodomain inhibitors typically show promiscuity between members of their family. We provide several lines of evidence that indicate that the reported effects of CREBBP/EP300 bromodomain inhibition are on target and not through inhibition of the BET family. First, we provide genetic evidence that disruption of CREBBP or EP300 bromodomains affects the proliferation of K562. Second, we found that CBP30 specifically downregulates the expression of genes bound by top levels of EP300 while JQ1 causes downregulation of genes associated with super-enhancers suggesting that the CBP30 effects are on target. Third, phenotypic effects of CBP30 treatment can be detected at concentrations as low as 0.5 µM. A previous report has estimated, taking into account the in vitro dissociation constants and cellular transcriptional responses to CBP30 and BET inhibitors, that treatment with CBP30 at concentrations lower than 3.3 µM are unlikely to affect the BET family [17]. Finally, the four unrelated CREBBP/EP300 bromodomain inhibitors cause similar phenotypic and gene expression effects. These effects are also shared by the potent catalytic inhibitor A-485. All these results strongly suggest that the observed effects are on target.

Our results indicate that the direct effect of CREBBP/EP300 bromodomain inhibition is blocking the ability of GATA1 and MYC to stimulate transcription by impairing the proper recruitment of CREBBP/EP300 to their binding sites and reducing the levels of H3K27ac (Fig. 7d). This in turn might prevent the recruitment of bromodomain-containing proteins such as BRD4 to target regulatory regions. Some of the affected regulatory regions are important enhancers that control the expression of *GATA1* or *MYC* and are critical for the proliferation of K562 cells [33]. Since GATA1 and MYC regulate their own transcription, downregulation of the expression of these transcription factors by CREBBP/EP300 bromodomain inhibitors is likely a consequence of their own impaired transactivation ability. These auto-inhibitory loops will further reinforce the effect of the inhibitors on target genes. Interestingly, MYC have been described to regulate the expression ribosomal genes and translation initiation factors [35] which is one of the main categories that we found downregulated by the inhibitors. GATA1 is a major regulator of chromatin accessibility in K562 cells [36], and it might facilitate the recruitment of MYC and HATs such as CREBBP/EP300 to its binding sites.

GATA1 is a multifaceted zinc finger transcription factor that is essential for the regulation of a set of genes related to the proliferation, differentiation and cell survival of erythroid progenitor cells. Inadequate GATA1 gene expression disturbs the balance of erythroid proliferation, survival and differentiation. Importantly, *GATA1* is highly expressed in K562 cells and is a critical transcription factor that mediates proliferation and chromatin accessibility in this cell line [33, 36]. Paradoxically, while structural mutations in *GATA1* that are found in almost all megakaryoblastic leukemia in patients with Down syndrome, overexpression of GATA1 has been reported in a subset of AML patients [37, 38] and high levels of GATA1 expression have been suggested to confer resistance to chemotherapy in acute megakaryocytic leukemia [39]. Further studies will be needed to evaluate the relevance of GATA1 overexpression in hematologic cancers.

Our study suggests that the sensitivity to CREBBP/EP300 inhibition can rely on multiple transcriptional programs existing in one given cell type rather that one single transcription factor. Given the large number of described interactions between CREBBP/EP300 and transcription factors, it is likely that CREBBP/EP300 bromodomain inhibitors can be effective in reducing the tumorigenesis of other cancer cell lines governed by other oncogenic transcription factors that depend on CREBBP/EP300 to stimulate transcription. Additionally, other histone acetyltransferases involved in supporting the expression of oncogenic transcription factors could be also candidates for therapeutic intervention.

## Conclusions

Our study shows that several hematologic cancer cell lines are sensitive to inactivation of CREBBP/EP300 bromodomains. Targeting CREBBP/EP300 bromodomains with small molecules displaces these histone acetyltransferases from chromatin reducing the levels of acetylation at critical regulatory elements and compromises cell proliferation. Our results suggest that CREBBP/EP300 bromodomain inhibitors might be able to reduce the tumorigenesis of cancers governed by oncogenic transcription factors that depend on CREBBP/EP300 to stimulate transcription and therefore hold therapeutic potential.

## Methods

### Cell lines and reagents

Human cancer cell lines K562, KMS11 and MM1S were purchased from ATCC. Antibodies were obtained from the following sources: EP300 (C-20) sc-585 from Santa Cruz, CREBBP (A-22) sc-369 from Santa Cruz, H3K27ac ab4729 from Abcam, MYC (N-262) sc-764 from Santa Cruz, GATA1 (N6) sc-265 from Santa Cruz, ACTB (Ac-15) A5441 from Sigma-Aldrich, PARP 9542 and Caspase3 9662 from Cell Signaling. CBP30 and I-CBP112 were purchased from Tocris Bioscience. C646 was purchased from Sigma-Aldrich. JQ1 was purchased from Selleck Chemicals. GNE-272 and CPI644 were synthesized in house. A-485 was obtained from the Structural Genomics Consortium.

### RNA interference, establishment of stable cell lines and proliferation assays

Non-inducible pGIZP and doxycycline inducible pTRIPZ vectors containing shRNAs against CREBBP or EP300, respectively, were purchased from Dharmacon. Target sequences were the following; shCREBBP TAAGTGATAATATTCATCC and shEP300 TTTCTTTGACTGTCCTGGA. Lentiviral infections were performed as previously described [40]. Stable K562 cell lines infected with shNT (nontarget) and shCREBBP were stablished after 2 weeks of selection with 2 µg/ml puromycin. Cells were re-infected with doxycycline inducible shEP300, and cells expressing RFP after the addition of doxycycline were sorted. Downregulation of CREBBP and EP300 was assessed by western blot after 6 days of treatment with 0.5 µg/ml doxycycline. For proliferation curves, cells were counted using a hemocytometer and plated at day 0 in triplicate for each condition and treated with 0.5 µg/ml doxycycline. To determine IC50 s, cells were grown in 96-well plates in the presence of increasing amounts of compound. At day seven, viability was determined using the CellTiter-Glow Luminescent Assay. IC50 values were calculated with a four-parameter variable-slope dose response curve using the GraphPad Prisms software.

### CRISPR-Cas9 gene editing and growth competition assays

gRNAs (Additional file 1: Table S1) were designed using the web tool cripsr.mit.edu with a quality score threshold above 80 to minimize off-target effects. Nontarget gRNAs sequences were previously described [41]. gRNAs were cloned into pKLV-U6gRNA(BbsI)-PGKpuro2ABFP (Addgene plasmid # 50946). Lentiviral particles were generated as previously described [40] and K562 cells previously modified to express Cas9 using pLentiCas9 Blast (Addgene plasmid # 52962) [42] were infected. Four days post-infection, growth competition assays were carried out by mixing an equal number of BFP +/gRNA expressing cells and non-gRNA transduced parental Cas9 expressing cells (BFP-). The percentage of BFP + cells was determined by flow cytometry at different days starting the day of the mixing (day 0) and the fold depletion of the percentage of BFP + cells compared to day 0 was calculated (d0%BFP +/dN  %BFP +). Introduction of mutations for each gRNA was confirmed by Sanger sequencing at day 4 post-infection. gRNAs targeting EP300 did not introduce mutations in CREBBP and vice versa. For the statistical analysis, the percentage of growth inhibition at day 14 compared to day 0 for each gRNA was calculated and adjusted to the percentage of growth inhibition of the nontarget gRNAs. The adjusted percentages of growth inhibition for each gRNA obtained in up to 4 independent experiments were pooled into categories (NT, 5′ coding region, non-conserved aminoacids of the bromodomain and conserved aminoacids of the bromodomain), and the categories were compared using the Tukey–Kramer test [43].

### Cell cycle analysis

Cell pellets were fixed with 70% ethanol in PBS at 4 °C for at least 1 h and stained with propidium iodide (100 ug/ml) in the presence of RNase A and 0.1% Triton X-100 at 4 °C for at least 30 min. Cell cycle distribution was measured using a BD LSRFortessa flow cytometer (BD Biosciences) and data analyzed using the FlowJo software. Three replicates were used per condition.

### RNA-seq

Cells were treated for 48 h, and total RNA was extracted using the RNeasy kit (Qiagen). Two biological replicates were used per condition. Library construction, sequencing, alignment to human genome hg19 transcript assembly and differential expression were done as previously described [44] using Nextpresso [45]. Genes changing expression with a FDR < 0.05 were considered as differentially expressed. For the detection GATA1 splicing variants, Cufflinks was run without annotation reference.

### Real-time qPCR

RNA was obtained as described above, cDNA synthesized using the SuperScript First-Strand Synthesis System (ThermoFisher) and real-time qPCR performed using the following primers GATA1.F GGATCCCGTGTGCAATGC, GATA1.R GGTCAGTGGCCGGTTCAC, MYC.F 5′AGGGTCAAGTTGGACAGTGTCA, MYC.R 5′TGGTGCATTTTCGGTTGTTG, CCND1.F CACGCGCAGACCTTCGTT, CCND1.R ATGGAGGGCGGATTGGAA, GAPDH.F GCACCGTCAAGGCTGAGAAC and GAPDH.R AGGGATCTCGCTCCTGGAA. Reactions were carried out in triplicate and expression levels normalized to GAPDH.

### Chromatin immunoprecipitation

Chromatin immunoprecipitation (ChIP) assays were performed according to the Millipore protocol. Cells were treated with 5 µM CBP30 and fixed with 2 mM DSG (Di(N-succinimidyl) glutarate for 45 min and 1% formaldehyde for 20 min. Cross-linking was stopped with 0.125 M glycine for 10 min, and chromatin was obtained and immunoprecipitated as previously described [44]. Immunoprecipitated chromatin was purified and used for qPCR amplification using the following oligonucleotides: GATA1_P.F 5′TCTCCCCCAAAGCCTGATC and GATA1_P.R 5′ CAGCTGGGAGTGGGCAGATA, MYC_P.F 5′GGTGGCAGAAGCCAGATCTC and MYC_P.R 5′GACCAGGGAGGCAAATGGA, CCND1_P.F 5′GCCTGTCCACTGGGAATCC and CCND1_P.R 5′AGCCCTCACTGGCATTCTCTT. HMGA2_P.F GAGTGGGCGGGTGAGAAAA and HMGA2_P.R GTTTGCATGCAGTGCAGTGA.

### Gene set enrichment analysis (GSEA)

For GSEAPreranked [28], genes were pre-ranked according to the statistic test of fold change for each treatment obtained in the RNA-seq analysis, setting ‘gene set’ as the permutation method and with 1000 permutations.

### ChIP-seq analysis

Super-enhancers and top EP300 occupied regions were identified using ROSE [7, 24]. Briefly, H3K27Ac and EP300 intervals were stitched together if they were within 12.5 kb and ranked by their ChIP-seq signal. Super-enhancers and top EP300 regions were mapped to the nearest gene using GREAT [46]. Metagene representations at regular enhancers and super-enhancers were calculated using bamToGFF (https://github.com/bradnerComputation/pipeline/blob/master/bamToGFF.py). Heat maps of ChIP-seq signals at given genomic locations were calculated using the Heat map tool from Galaxy Cistrome [47]. ChIP-seq data were visualized at the UCSC genome browser using the hg19 human genome build [48].

### Statistical methods

The enrichment of differentially expressed genes in super-enhancers or top EP300 associated genes was calculated according to Xi-squared test. Benjamini *p* values for gene ontology were calculated using DAVID [49, 50].

### Source of public data

GATA1 gene expression was obtained from The Cancer Cell Line Encyclopedia (CCLE) website (http://www.broadinstitute.org/ccle/home) and from the TCGA cBioPortal website (http://www.cbioportal.org) [51]. ChIP-seq data were from the ENCODE project [52] and downloaded from the UCSC Genome Browser website (http://genome.ucsc.edu) and have the following GEO accessions numbers: H3K27ac (GSM733656) and EP300 (GSM935401), GATA1 (GSM1003608) and MYC (GSM935516).

## Additional files

**Additional file 1: Table S1.** gRNAs used for gene editing. **Fig. S1.** Statistical analysis of the CRISPR-Cas9 growth competition experiments. Tukey Kramer analysis of the adjusted percentages of growth inhibition caused by gRNAs targeting different regions of CREBBP (A) or EP300 (B). 5′ coding region (5′), non-conserved aminoacids of the bromodomain (ncBD), conserved aminoacids of the bromodomain (cBD) and non-target (NT). **Fig. S2.** Enrichment of gene expression changes after treatment with CBP30 and I-CBP112. (A) p-values for enrichment of SE-associated genes (SE) and genes with top levels of EP300 (EP300) in genes upregulated and downregulated by CBP30 and I-CBP112 treatments. (B) GSEA analysis of changes in gene expression caused by the indicated treatments and gene sets. **Fig. S3.** GATA1 mRNA expression in cancer cell lines and patients. (A) mRNA levels of GATA1 determined by microarray in CCLE lines grouped by cancer type. (B) GATA1 mRNA levels determined by RNAseq in cancer patients according to TCGA. **Fig. S4.** Expression of GATA1 splicing variants in K562 (A) Three variants are expressed in K562 according to the analysis of the RNA-seq experiment (B) Graph shows the levels of expression of the different variants in K562 cells treated with vehicle or two concentrations of CBP30. P-values for significant changes (p ≤ 0.05) are shown. **Fig. S5.** Human myeloma cell lines with MYC amplifications or translocations are sensitive to CBP30. (A) IC50s of growth inhibition in KMS11 or MM1S cells treated with JQ1, C646 and CBP30 for 7 days. (B) mRNA (upper panel) and protein (lower panel) levels of MYC in KMS11 or MM1S cells treated with 2 µM CBP30, 10 µM C646 and 150 nM JQ1 for 48 hours.

**Additional file 2: Table S2.** Genes downregulated by the indicated treatments. Presence of super-enhancers and top EP300 occupancy is also indicated.
