# Supplementary material for: CREBBP/EP300 bromodomains are critical to sustain the GATA1/MYC regulatory axis in proliferation
Source: Epigenetics Chromatin. 2018 Jun 8;11:30. doi: 10.1186/s13072-018-0197-x (PMC5992658; doi:10.1186/s13072-018-0197-x)

SUPPLEMENTARY TABLES AND FIGURES_ Garcia-Carpizo et al.

Supplementary Table 1. gRNAs used for gene editing.

Supplementary Table 2. Genes downregulated by the indicated treatments. Presence of super-enhancers and top EP300 occupancy is also indicated.

Supplementary Figure 1. Statistical analysis of the CRISPR-Cas9 growth competition experiments. Tukey Kramer analysis of the adjusted percentages of growth inhibition caused by gRNAs targeting different regions of CREBBP (A) or EP300 (B). 5’ coding region (5’), non-conserved aminoacids of the bromodomain (ncBD), conserved aminoacids of the bromodomain (cBD) and non-target (NT).

Supplementary Figure 2. Enrichment of gene expression changes after treatment with CBP30 and I-CBP112. (A) p-values for enrichment of SE-associated genes (SE) and genes with top levels of EP300 (EP300) in genes upregulated and downregulated by CBP30 and I-CBP112 treatments. (B) GSEA analysis of changes in gene expression caused by the indicated treatments and gene sets.

Supplementary Figure 3. GATA1 mRNA expression in cancer cell lines and patients. (A) mRNA levels of GATA1 determined by microarray in CCLE lines grouped by cancer type. (B) GATA1 mRNA levels determined by RNAseq in cancer patients according to TCGA.

Supplementary Figure 4. Expression of GATA1 splicing variants in K562 (A) Three variants are expressed in K562 according to the analysis of the RNA-seq experiment (B) Graph shows the levels of expression of the different variants in K562 cells treated with vehicle or two concentrations of CBP30. P-values for significant changes ($p \leq 0.05$) are shown.

Supplementary Figure 5. Human myeloma cell lines with MYC amplifications or translocations are sensitive to CBP30. (A) IC50s of growth inhibition in KMS11 or MM1S cells treated with JQ1, C646 and CBP30 for 7 days. (B) mRNA (upper panel) and protein (lower panel) levels of MYC in KMS11 or MM1S cells treated with 2µM CBP30, 10 µM C646 and 150 nM JQ1 for 48 hours.

Supplementary Table 1

gRNA ID	Gene	Location	Sequence
37	CREBBP	5'Region	GGGCAGGCGCAAGTCATGAA
38			GATGAGCTGATACCCAATGG
39		BD non conserved	GGCAGTACGTGGACGACGTC
40			CTCCACCATCAAGCGGAAGC
41			GGATCTACAGGCTGCCGGAA
63		BD Conserved	TTGATGGTGGAGAGGTCCAT
64			TGACATCGTAAAGAATCCCA
65			TGTCTTGCGATTATAGAGCC
47	EP300	5'Region	GGCACTAGTGGACCAAATCA
48			GGGTAGTCCAAGAGCTGCAT
49		BD non conserved	GGGTCCACAGGTTGACGAAA
50			ATATCATCGACATACTGCCA
51			TGTTTTCCGGTTATATAACC
59		BD Conserved	TGATATTGTGAAGAGCCCCA
60			TAATGGTAGAAAGATCCATG
61			TTAATGGTAGAAAGATCCAT
62			ATAATGCCTGGTTATATAAC
22	Non Target	None	GTCGTGAAGTGCATTCGATC
23			GTGTATCTCAGCACGCTAAC
6	BRD4	BD1	GGGAACAATAAAGAAGCGCT
31		5' Region	AGATTTCTCAATCTCGTCCC
32			GATTTCTCAATCTCGTCCCA

Supplementary Fig1

A

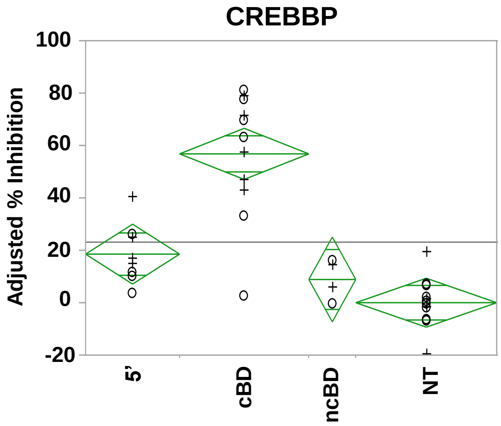

Means Comparisons

Comparisons for all pairs using Tukey-Kramer HSD

Ordered Differences Report

Level	- Level	Difference	Std Err Dif	Lower CL	Upper CL	p-Value
Cons AA, BRD	Non-Target	56.80264	6.618424	38.8398	74.76553	<.0001 *
Cons AA, BRD	Non-Cons AA, BRD	47.93802	9.257568	22.8123	73.06374	<.0001 *
Cons AA, BRD	5'Reg	38.26775	7.367381	18.2721	58.26335	<.0001 *
5'Reg	Non-Target	18.53489	7.236974	-1.1068	38.17657	0.0699
5'Reg	Non-Cons AA, BRD	9.67027	9.709420	-16.6818	36.02235	0.7528
Non-Cons AA, BRD	Non-Target	8.86462	9.154129	-15.9804	33.70959	0.7682

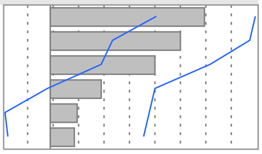

B

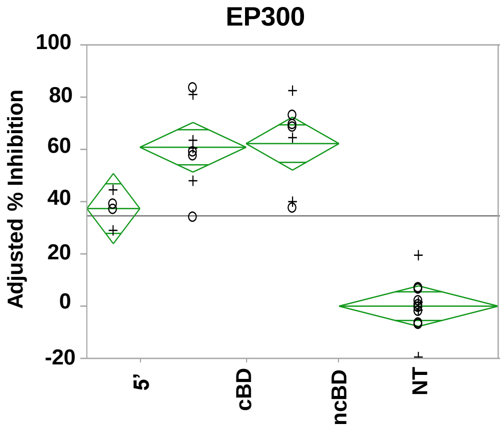

Means Comparisons

Comparisons for all pairs using Tukey-Kramer HSD

Ordered Differences Report

Level	- Level	Difference	Std Err Dif	Lower CL	Upper CL	p-Value
Non-Cons AA, BRD	Non-Target	62.21842	6.226934	45.1780	79.25883	<.0001 *
Cons AA, BRD	Non-Target	60.81556	5.976081	44.4616	77.16949	<.0001 *
5'Reg	Non-Target	37.34406	7.559211	16.6578	58.03034	0.0002 *
Non-Cons AA, BRD	5'Reg	24.87435	8.206433	2.4169	47.33179	0.0258 *
Cons AA, BRD	5'Reg	23.47150	8.017754	1.5304	45.41260	0.0327 *
Non-Cons AA, BRD	Cons AA, BRD	1.40286	6.776239	-17.1408	19.94647	0.9968

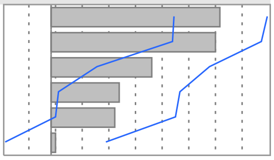

Supplementary Fig2

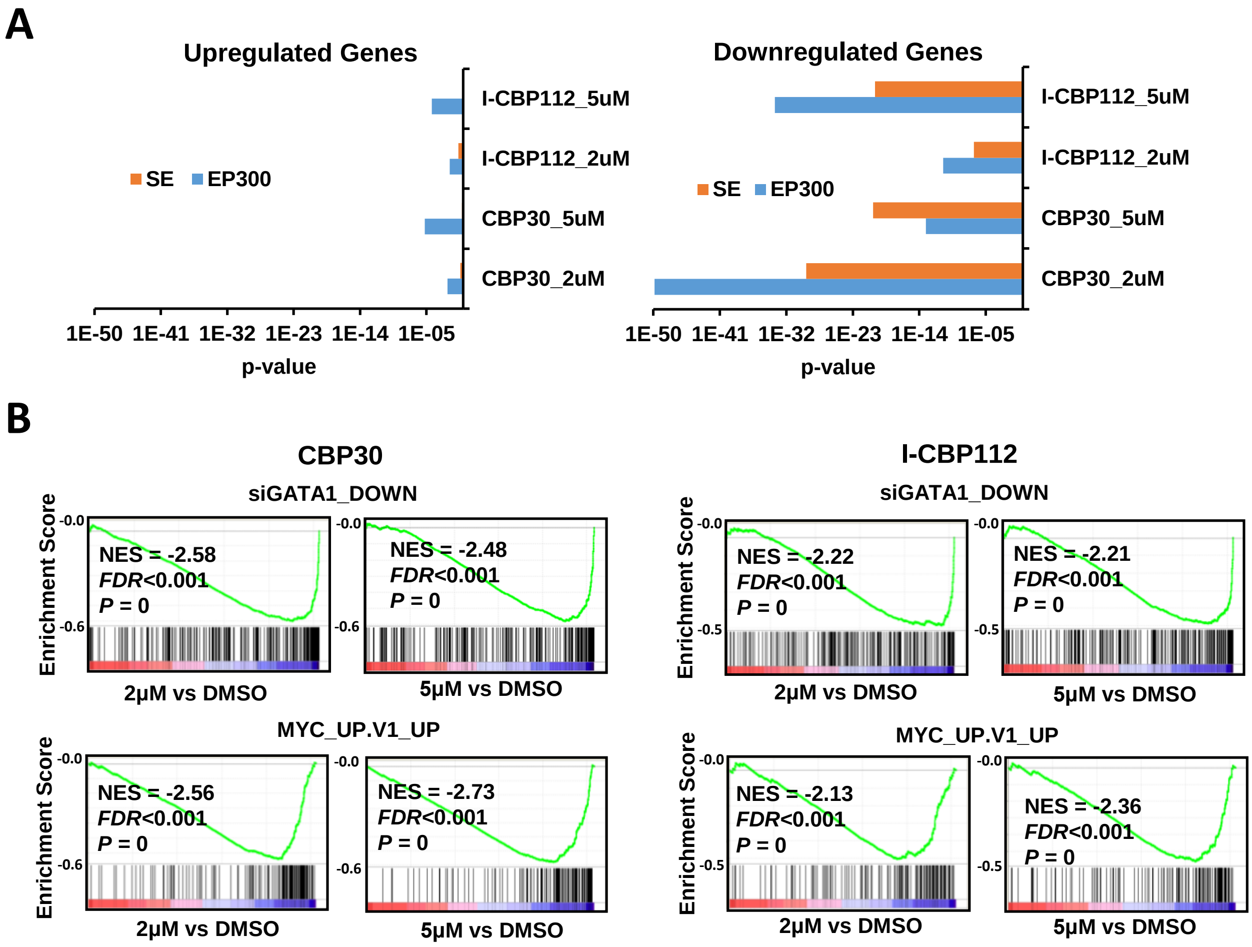

Supplementary Fig3

A

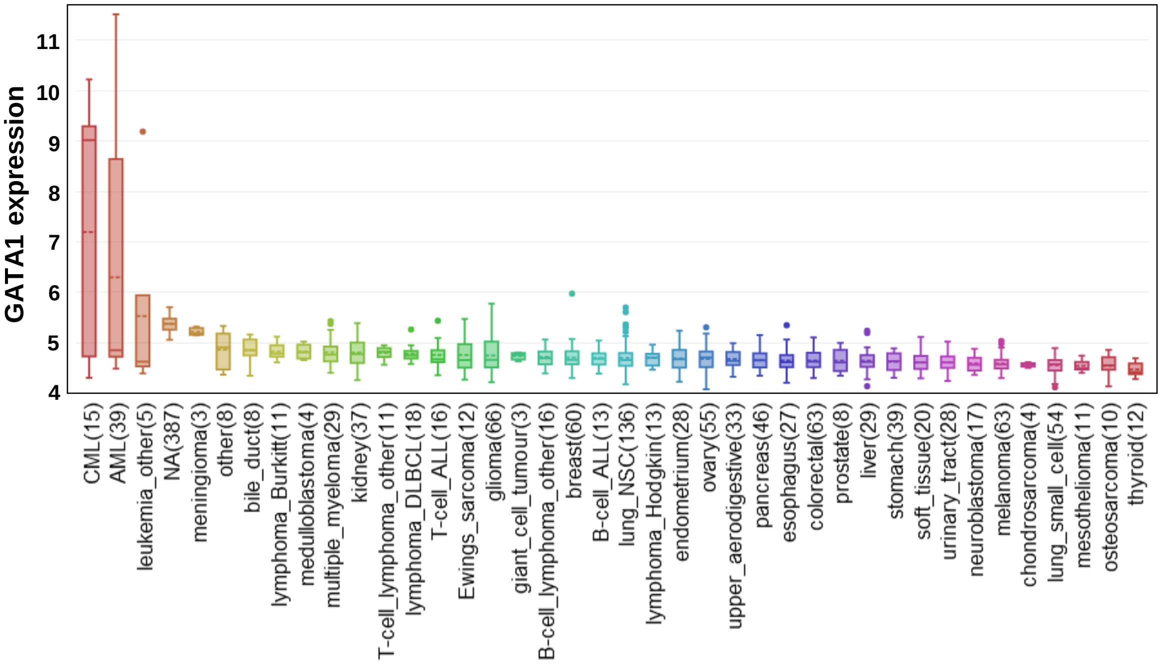

B

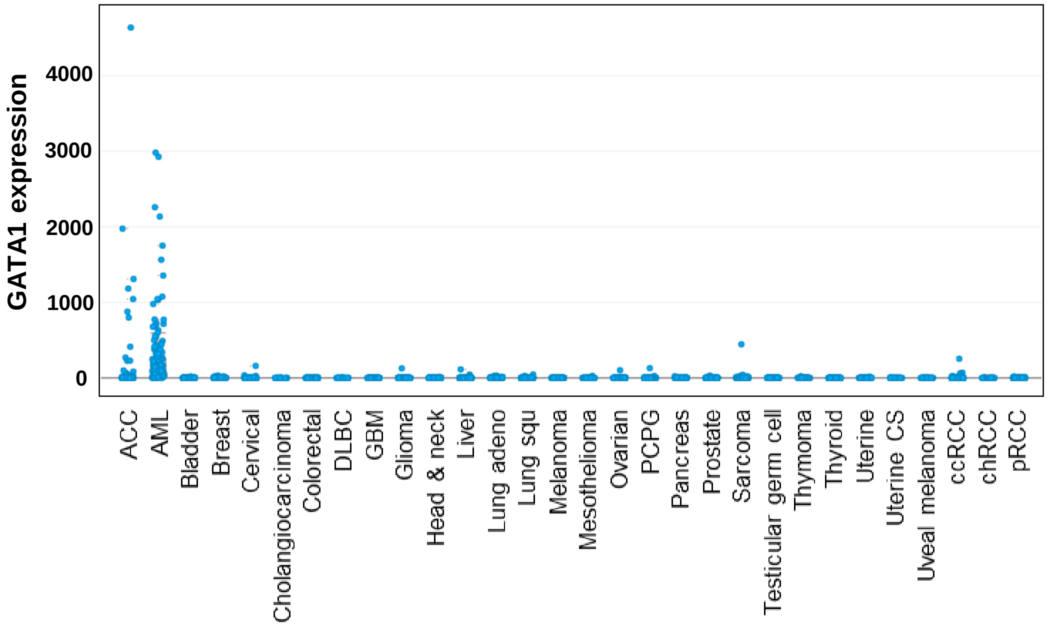

Supplementary Fig4

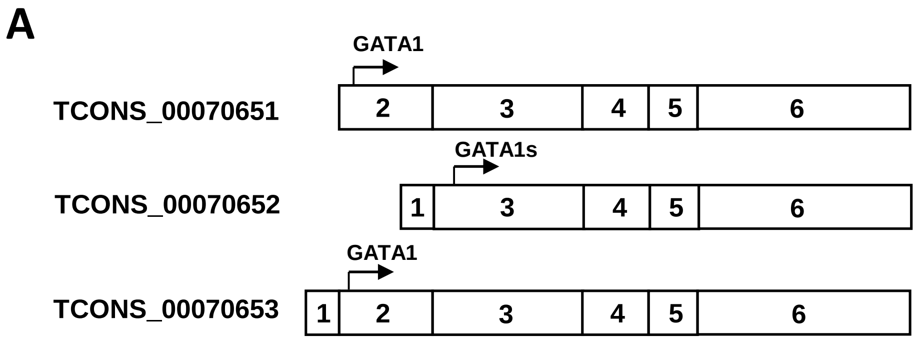

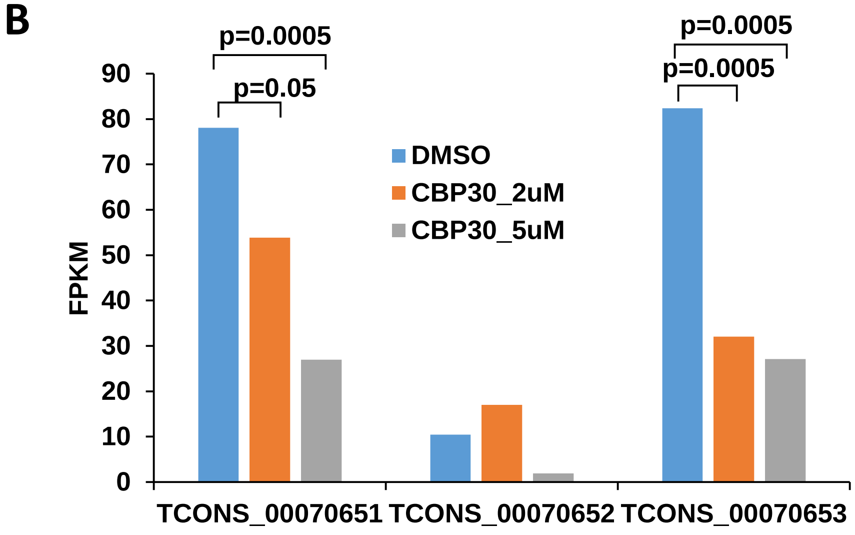

Supplementary Fig 5

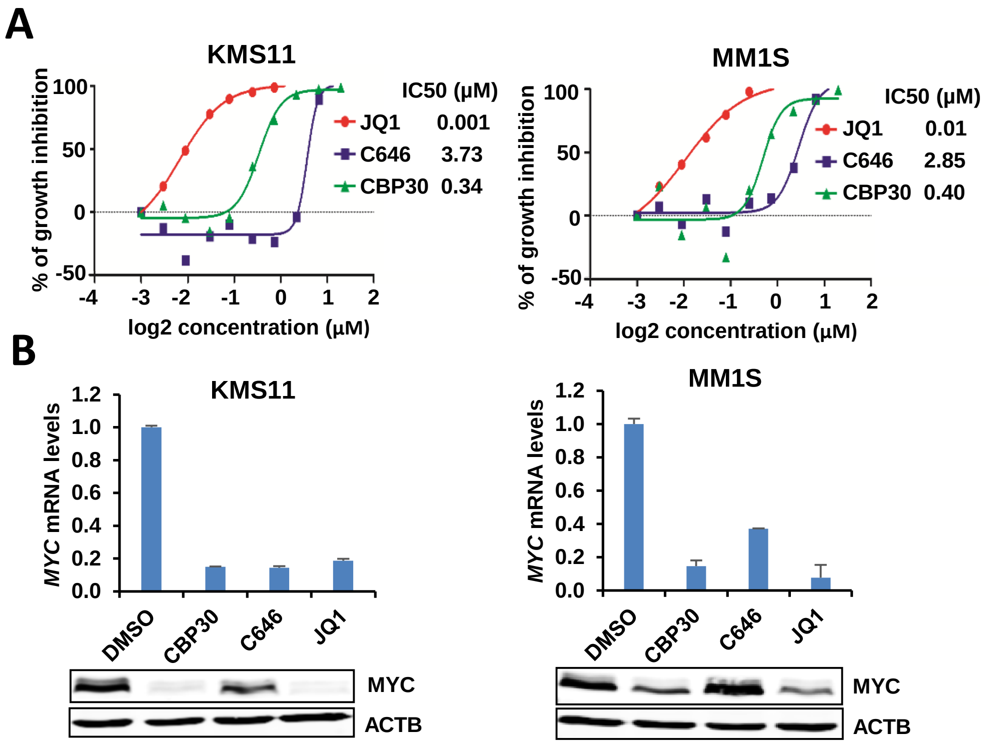

Supplement: Supplementary file 1 — Additional file 1: Table S1. gRNAs used for gene editing. Fig. S1. Statistical analysis of the CRISPR-Cas9 growth competition experiments. Tukey Kramer analysis of the adjusted percentages of growth inhibition caused by gRNAs targeting different regions of CREBBP (A) or EP300 (B). 5′ coding region (5′), non-conserved aminoacids of the bromodomain (ncBD), conserved aminoacids of the bromodomain (cBD) and non-target (NT). Fig. S2. Enrichment of gene expression changes after treatment with CBP30 and I-CBP112. (A) p-values for enrichment of SE-associated genes (SE) and genes with top levels of EP300 (EP300) in genes upregulated and downregulated by CBP30 and I-CBP112 treatments. (B) GSEA analysis of changes in gene expression caused by the indicated treatments and gene sets. Fig. S3. GATA1 mRNA expression in cancer cell lines and patients. (A) mRNA levels of GATA1 determined by microarray in CCLE lines grouped by cancer type. (B) GATA1 mRNA levels determined by RNAseq in cancer patients according to TCGA. Fig. S4. Expression of GATA1 splicing variants in K562 (A) Three variants are expressed in K562 according to the analysis of the RNA-seq experiment (B) Graph shows the levels of expression of the different variants in K562 cells treated with vehicle or two concentrations of CBP30. P-values for significant changes (p ≤ 0.05) are shown. Fig. S5. Human myeloma cell lines with MYC amplifications or translocations are sensitive to CBP30. (A) IC50s of growth inhibition in KMS11 or MM1S cells treated with JQ1, C646 and CBP30 for 7 days. (B) mRNA (upper panel) and protein (lower panel) levels of MYC in KMS11 or MM1S cells treated with 2 µM CBP30, 10 µM C646 and 150 nM JQ1 for 48 hours. [file 13072_2018_197_MOESM1_ESM.pdf]
